# Effect of Surgeon Service Volume on Survival of Liver Transplant Patients: A Nationwide Cohort Study

**DOI:** 10.3390/jcm15010321

**Published:** 2026-01-01

**Authors:** Liang-Yu Chiang, Tzu-Wei Wang, Pei-Tseng Kung, Wen-Chen Tsai

**Affiliations:** 1 Department of Orthopedic Surgery, Taichung Armed Forces General Hospital, Taichung 41152, Taiwan; jazking68@gmail.com; 2 Department of Public Health, China Medical University, Taichung 406040, Taiwan; 3 School of Medicine, National Defense Medical University, Taipei 11490, Taiwan; 4 Department of Health Services Administration, China Medical University, No. 100, Sec. 1, Jingmao Rd., Beitun District, Taichung 406040, Taiwan; vicky840503@gmail.com; 5 Department of Healthcare Administration, Asia University, Taichung 413305, Taiwan; ptkung@asia.edu.tw; 6 Department of Medical Research, China Medical University Hospital, China Medical University, Taichung 406040, Taiwan

**Keywords:** surgeon service volume, cumulative service volume, physician service volume, liver transplant, survival rate

## Abstract

**Background/Objectives**: Liver transplantation is an effective treatment for end-stage liver disease, and patients treated by surgeons with higher service volumes have better therapeutic outcomes. However, few studies have examined the effects of cumulative service volume on the survival of liver transplant patients. The objective of this study was to investigate the effect of a surgeon’s cumulative service volume on the survival rates of liver transplant patients. **Methods**: The study was a retrospective and nationwide cohort study. Patients who underwent a liver transplant in 2005–2013 were identified. The data were from the Taiwan National Health Insurance Research Database. The primary outcome was the effect of surgeon service volume on 1-year survival after surgery for liver transplant patients. **Results**: A total of 3233 patients who underwent liver transplantation had a first-year survival rate of 85.8%. The high relative service volume group (>307 cases) had the highest patient survival rate at 1 year after operation (95.31%), while the low relative service volume group (<31 cases) had the lowest survival rate (71.39%). After relevant adjustment variables, the risk of mortality was significantly higher among patients operated on when their surgeons had accumulated fewer than 41 prior transplant cases, and the risk of mortality decreased as the cumulative service volume of surgeons rose. **Conclusions**: This nationwide cohort study demonstrated an association, rather than a causal relationship, between surgeon cumulative service volume and 1-year survival after liver transplantation. One-year survival reached approximately 85% once surgeons had accumulated 41–60 prior transplant cases. These findings may provide a reference for understanding the clinical learning curve in liver transplantation.

## 1. Introduction

According to the Taiwanese Ministry of Health and Welfare, in 2016, liver and intrahepatic cholangiocarcinoma were the second leading cause of malignant tumors, with a mortality rate of 35.5 per 100,000. Chronic liver disease and cirrhosis is the tenth leading cause of death, with a mortality rate of 20.1 per 100,000 [1]. Liver disease is common in Taiwan and liver transplantation is considered the most effective treatment to help end-stage liver disease patients return to normal daily life [2,3,4].

Although liver transplantation can prolong the life of patients with liver disease, according to the Central Health Insurance Department, the 1-year, 3-year, and 5-year survival rates in patients after surgery were only 86%, 78%, and 74%, respectively [5]. In addition, previous studies have reported that some patients develop serious postoperative complications, such as bile leakage, hepatic artery thrombosis, intra-abdominal hemorrhage, organ rejection, and viral infection, resulting in recurrent hospitalization or re-transplantation [6,7], which in turn led to increasing medical costs. Furthermore, bleeding and intraoperative blood transfusion also impact the postoperative survival of patients with hepatocellular carcinoma [8]. Previous studies have suggested that the cause of bleeding may be associated with the physician’s professional competence and medical experience, both of which are important factors in treatment success [9].

Treatment outcomes are closely associated with surgeon service volume. In 1973, Adams and colleagues conducted the first study on the relationship between physician service volume and treatment outcomes in their analysis of the rates of complications of coronary arteriography [10]. Since then, similar studies have been performed on gastric cancer resection in Taiwan [11], colorectal surgery [12], and bariatric surgery [13] in the United States, as well as an Italian report on results in 26 clinical areas [14]. Most studies have demonstrated that physicians with higher service volume have better treatment outcomes than those with lower service volume [15,16].

According to previous studies, the factors influencing the survival of liver transplant patients include: the service volume of the physician; the patient’s gender, age, education, marital status, and economic and environmental factors, health status and health behavior; the characteristics of the surgical hospital; source of organ donation; and postoperative medication control [17,18,19,20,21,22,23,24,25,26,27,28]. Burroughs et al. studied the effect of physician service volume on European liver transplant patients and showed that the mortality rate of patients in the high service volume group (≥70 cases) was lower at 3 months and 1 year after surgery, compared with patients in the medium service volume group (37–69 cases) and those in the low service volume group (36 cases) [29].

While past studies have focused on physician service volume and treatment outcomes, none have determined the exact number of operations in a year of a surgeon’s service that corresponds with the optimal survival of liver transplant patients. Therefore, this study aimed to investigate the effect of surgeon service volume (both relative and cumulative) and related factors on patient survival in the first year after liver transplantation.

## 2. Materials and Methods

### 2.1. Data Sources and Study Populations

This study was a retrospective cohort study. Data from the National Health Insurance Research Database for 2000–2014 were provided by the Ministry of Health and Welfare, and the Cause of Death Database for 2005–2014 was used to determine the cause of death. In this study, we selected patients who underwent liver transplantation from 2005 to 2013 and were coded 50.5 on the International Classification of Diseases, Ninth Revision, Procedure Coding System (ICD-9-PCS). Patients with two or more liver transplants (39 patients) were excluded, resulting in a total of 3233 patients identified. No organs from executed prisoners were included in this study. The study was approved by the hospital Institutional Review Board (CMUH107-REC3-033; approval date: 27 September 2018), which waived the requirement for informed consent.

### 2.2. Definition of Relevant Variables

The survival of the patient within 1 year after liver transplantation is influenced by the technique and experience of the surgeon; therefore, previous studies considered whether the patient died within the first year after liver transplantation as a dependent variable [30,31]. In this study, each subject was followed up for 1 year after liver transplantation and was categorized as having died (confirmed in the Cause of Death Data statistical database) or living. The independent variable was the surgeon service volume. Since the National Health Insurance Research Database has been available only since 2000, the amount of surgeon service volume was calculated beginning 1 January 2000. The surgeon service volume was defined as the number of liver transplants performed by the primary operating surgeon since 1 January 2000 up to the time when the patients underwent liver transplants, and included both relative and cumulative service volume. In procedures involving multiple surgeons, surgeon service volume was attributed to the primary surgeon recorded in the National Health Insurance database, as only one surgeon is registered for reimbursement purposes. Relative service volume was divided by quartiles, with low surgeon service volume at ≤25%, medium volume at 25–75%, and high volume at ≥75%. Cumulative service volume was determined by cases as follows: ≤20, 21–40, 41–60, 61–80, 81–100, 101–120, 121–140, 141–160, 161–180, 181–200, 201–230, 231–260, 261–300, 301–400, 401–500, and ≥501 cases, in order to locate the group at which cumulative service volume provided optimal patient survival in the 1-year period after liver transplantation.

Control variables were patient characteristics (gender, age), economic factors (monthly salary), health status (severity of comorbidity), environmental factors (degree of urbanization of the residential area), surgeon characteristics (whether the surgeon changed the practice hospital), surgical hospital characteristics (hospital accreditation level, hospital ownership), and year of operation. Patient age was divided into six groups of <20 years, 20–34 years, 35–44 years, 45–54 years, 55–64 years, and ≥65 years old. Monthly salary was divided into six groups of <564 United States dollars (USD), 564–744 USD, 745–940 USD, 941–1186 USD, 1187–1496 USD, and >1497 USD. Severity of comorbidity was determined by using the Charlson Comorbidity Index (CCI), which uses ICD-9-CM codes for 17 diseases to calculate the cumulative comorbidity of patients; it is the most widely used such tool [32]. The primary and secondary diagnoses recorded for the outpatient and inpatient visits for each patient for the two years prior to the liver transplant were converted into numerical scores, the liver-related diagnoses were excluded, and the total score was then calculated as the severity of comorbidity. Codes were calculated and divided into three groups: 0, 1–2, and ≥3. The degree of urbanization of the residential area was used to denote environmental factors. According to Liu et al., in 2006, the 359 townships in Taiwan are divided into seven levels (high degree of urbanization, moderate degree of urbanization, emerging town, general township urban area, old age town, agricultural town, and remote township), as a 7-cluster of urbanization [33].

Other characteristics included whether the surgeon changed the practicing hospital, defined as the surgeon changing the practice hospital within the two years before the liver transplant operation (yes/no). The characteristics of the surgical hospital included hospital accreditation level (medical center or regional hospital) and hospital ownership (public hospital or non-public hospital). The hospital where the patients underwent liver transplant surgery was defined as a surgical hospital for patients. The patients were examined according to year of transplantation (all years from 2005 to 2013, inclusive) to control for potential factors that could not be observed between years.

### 2.3. Statistical Analysis

Log-rank tests were used to perform unadjusted comparisons of survival curves across categories of surgeon service volume (relative and cumulative), patient characteristics, economic factors, health status, environmental factors, surgeon characteristics, surgical hospital characteristics, and year of transplantation, with patient mortality in the 1-year period after liver transplantation. The Cox Proportional Hazard Model was used to investigate the effects of different surgeon service volumes on the mortality of patients at 1 year after liver transplantation and associated risk factors. This study represented statistically significant differences at *p* < 0.05. Cox survival curves were used to depict the survival curve of patients at 1 year after liver transplant by surgeon service volume, after adjusting for other variables. SAS version 9.4 (SAS Institute Inc., Cary, NC, USA) statistics software was used for data processing and statistical analysis.

## 3. Results

Table 1 shows the distribution of the characteristics of the 3233 study subjects included in the statistical analysis. For relative surgeon service volume by quartile, low surgeon service volume was ≤31 cases, medium volume was 32–306 cases, and high volume was ≥307 cases. In all the 16 Surgeon Cumulative Service Volume categories, most patients were in the lowest volume category, i.e., 619 or 19.15% of all cases. The number of survivors at 1 year after liver transplantation was 2774, or 85.80%; the number of survivors at 3 years was 2595, or 80.27%.

**Figure 1:**
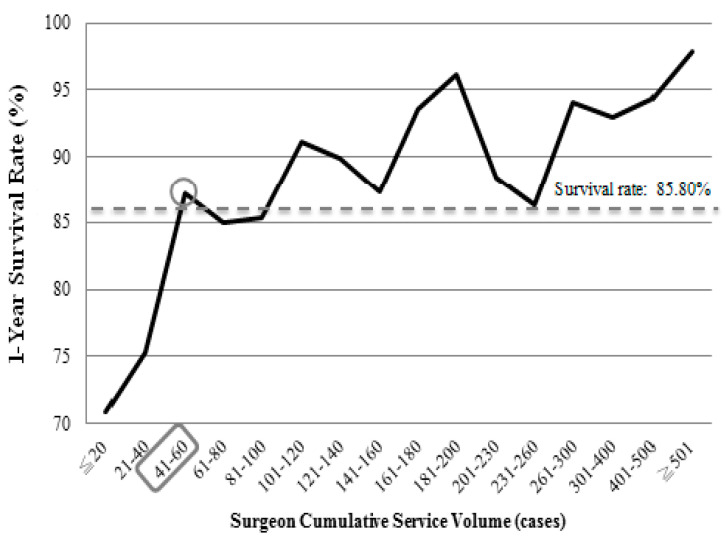
Survival rates of the patients within 1 year after liver transplantation according to surgeon cumulative service volume from 2005–2013.

Table 2 shows that surgeon service volume, comorbidity severity, hospital accreditation level, hospital ownership, and year of transplantation were significantly associated with the 1-year survival status of liver transplant patients (*p* < 0.05). In terms of relative service volume, the higher the surgeon’s service volume, the better the patient’s survival rate at 1 year after the operation. The high service volume group (≥307 cases) had the highest patient survival rate at 1 year after operation (95.31%), while the low service volume group (≤31 cases) had the lowest (71.39%). We further analyzed cumulative service volume. When the cumulative service volume of surgeons was ≤20 cases, the 1-year survival rate of liver transplant patients was 70.92%; for a cumulative service volume of 21–40 cases, the 1-year survival rate was 75.31%. When the surgeon’s cumulative service volume was 41–60 cases, the 1-year survival rate was 87.15%. After this point, the survival rate of liver transplant patients remained stable and gradually increased. The 1-year survival rates of patients with surgeons with a cumulative service volume of ≥60 cases were higher than those with 41–60 cases, as shown in Figure 1.

In terms of comorbidity severity, patients with higher comorbidity scores had lower rates of 1-year survival. Patients with a CCI score of 0 had the highest 1-year survival rate (88.59%), while those with a CCI score of ≥3 had the lowest (82.82%). In terms of hospital accreditation, patients treated at a medical center (86.01%) had a higher 1-year survival rate than those treated at a regional hospital (78.72%). For hospital ownership, patients at non-public hospitals had a greater 1-year survival rate (86.67%) than patients at public hospitals (83.93%). For year of transplantation, survival rates increased each year. In 2006, the 1-year survival rate was the lowest, 77.62%, and in 2013, 90.34% was the highest. However, survival rates did not differ by patient gender, age, monthly salary, urbanization of residence area, or whether the surgeon changed the practice hospital (*p* > 0.05).

**Figure 2:**
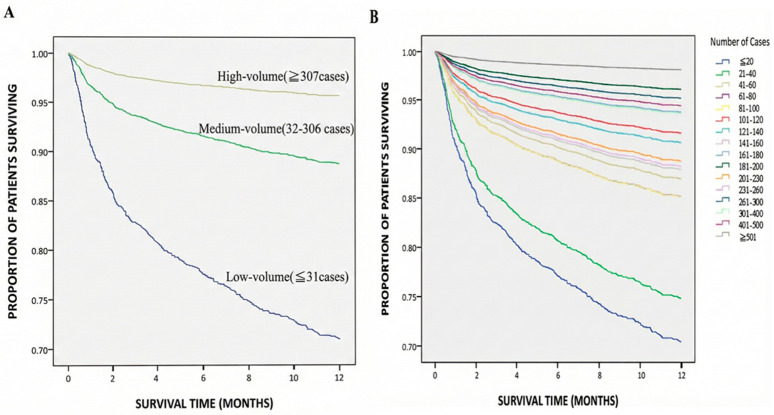
The survival curves of the patients within 1 year after liver transplantation from 2005 to 2013 based on (**A**) surgeon relative service volume (medium volume: HR 0.35, 95% CI 0.28–0.43; high volume: HR 0.13, 95% CI 0.09–0.19; both *p* < 0.001) and (**B**) surgeon cumulative service volume.

After adjusting for the relevant variables, we looked at which variables were associated with risk of mortality at 1 year (Table 3). Both surgeon service volume and comorbidity severity were significantly associated with the risk of mortality in patients 1 year after liver transplantation (*p* < 0.05). Figure 2 shows the 1-year patient survival curve after liver transplantation from 2005–2013 for patients according to surgeon relative service volume and surgeon cumulative service volume, respectively.

In terms of surgeon service volume, surgeons were divided into the low, medium and high group by the number of liver transplants performed. Using the lowest quartile group (≤31 cases) as the reference group (Adjusted Model 1, Table 3), we found that patients treated when surgeons’ accumulated service volume fell into the middle group (32–306 cases) or the high group (≥307 case) had statistically significantly lower risk of mortality, with hazard ratio (HR) values of 0.35 (95% confidence interval [CI]: 0.28–0.43; *p* < 0.001) and 0.13 (95% CI: 0.09–0.19; *p* < 0.001), respectively.

We further focused on the number of liver transplants performed by the surgeon, measured as the cumulative service volume. When this study used ≤20 cases (lowest) of cumulative surgeon service as the reference group (Adjusted Model 2, Table 3), the risk of mortality of liver transplant patients decreased as the cumulative service volume of surgeons rose, especially the cumulative service volume higher than 40 cases (*p* < 0.001). This result confirmed that the survival of patients with liver transplants improved and remained steadily better once the cumulative service volume of surgeons reaches 41–60 cases (Table 3).

In terms of severity of comorbidity, with CCI = 0 as the reference group, the risk of mortality in liver transplant patients rose, as did the comorbidity score. For example, the risk of mortality in liver transplant patients with CCI ≥ 3 was 1.54-fold (95% CI: 1.14–2.08) higher than that of those with CCI of 0 (*p* = 0.005). However, other variables had no effect on 1-year mortality (*p* > 0.05). Patients who received a liver transplant from a surgeon who did not change the practice hospital had a lower risk of mortality than those whose surgeon did change hospitals within two years before surgery (HR = 0.77, 95% CI: 0.24–2.50), but the difference was not statistically significant (*p* = 0.668).

## 4. Discussion

The results of this study indicate that the higher the surgeon’s service volume, the lower the risk of mortality in patients 1 year after liver transplantation. In a previous US study that divided the number of liver transplants performed by surgeons in a given year into low (<3 cases), medium (3–9 cases) and high (>9 cases), service volume was significantly associated with postoperative mortality and length of hospitalization. The postoperative mortality rate was 3.21-fold higher in the low service group than in the high service group, and the postoperative mortality rate was 2.81-fold greater in the medium service group than in the high service group [34]. Their results were consistent with the findings in our study. In the study by Burroughs et al., the number of cases of liver transplantation performed in Europe from 1988 to 2003 was collected; the mortality rate was measured at 3 months and 1 year after surgery, and the service volume was divided into three groups: high (≥70 cases), medium (37–69 cases) and low (≤36 cases). The patients in the high service volume group had the lowest mortality rates at 3 months and 1 year after surgery [29]. Their finding also corresponded with our study. In addition, both US and Taiwanese studies found that surgeons with higher service volume of organ transplants have better postoperative outcomes than those with lower service volume. The result may be related to the skill or experience of the surgeon [35,36,37,38,39].

In the past, many studies of physician service volume and treatment outcomes have divided the physician service volume into low (≤25%), medium (25–75%), and high (≥75%). In terms of service volume, relative volume may be less important than the cumulative service volume of the physician. We assumed that the cumulative service volume of the surgeon could well be the critical factor influencing the survival of patients within 1 year after liver transplantation. In this study, analysis of the surgeon service volume included both relative and cumulative service volume. At first, we analyzed the results of surgeons with 5 or 10 cases per group, but the 1-year survival rate of the patients was inconsistent. We then extended the cumulative service volume to 20 cases per group, and the survival rate of the patients was relatively steady after the surgery. Therefore, we analyzed the cumulative service volume of surgeons treating 200 cases or fewer by groups of 20 cases. Our results showed that when the cumulative service volume of surgeons reached 41–60 cases, the 1-year survival rate of liver transplant patients was 87.15%, which was higher than the overall 1-year survival rate of 85.80%. The 1-year survival rate of liver transplant patients increased as the surgeon service volume rose beyond 41–60 cases. Therefore, when the surgeon’s cumulative service volume reaches 41–60 cases, the surgeon’s liver transplant technique may be established enough to maximize their patients’ likelihood of the survival.

Among the variables influencing the survival of patients 1 year after liver transplantation, severity of comorbidity was also analyzed in a previous study in Taiwan of 1876 liver transplant patients. The researchers found that patients with hepatitis C after liver transplantation had higher rates of complications of diabetes mellitus and lower survival rates [40]. A previous US study suggested that the risk of postoperative transplantation failure in liver transplant patients with comorbidity scores CCI ≥ 6 was 3.95-fold higher than that of liver transplant patients with CCI scores < 6 [41]. In addition, the study of Pischke et al. in Germany tracked the 15-year survival of 114 patients with liver transplants and found higher rates of mortality and transplant failure in patients with BMI ≥ 24 kg/m^2^ compared to those with BMI < 24 kg/m^2^ [42]. Diseases such as hypertension, hyperlipidemia, diabetes, and obesity can lead to cardiovascular disease, which is a significant factor for mortality in liver transplant patients [22]. The above studies all indicate that patients with poor health status and more comorbidities before liver transplantation have a higher risk of postoperative mortality. Their findings were consistent with the results of our study.

The medical team performing the liver transplant surgery is also a very important factor in determining treatment outcomes after surgery. However, no research has yet been conducted on this factor. We analyzed the effect on patient survival of surgeons changing their medical team (by moving to another hospital) in the 2 years prior to the surgery. The results showed that patients of surgeons who did not change their hospitals had a lower risk of mortality than patients of those who did (HR = 0.77, *p* = 0.668), but the difference was not statistically significant. This result may be due to the small number of patients whose surgeons did change their hospitals; only eight patients received surgery from five surgeons who had changed their practice hospital.

In order to control for the potential factors that may not be observed in each year that affect patient survival after liver transplantation, we analyzed the effect of the year of transplantation. The results of the study showed that, in the multivariate analysis (Cox Proportional Hazard Model), the risk of mortality was higher but not significantly different in patients at 1 year after liver transplantation in 2006 and 2007 (*p* > 0.05). In the log-rank test, the survival rate of patients who underwent liver transplantation in 2006 and 2007 was significantly lower (*p* < 0.05), or 77.62% and 79.53%, respectively. One reason may be that the guidelines for the standard tumor size of liver transplant after 2006 changed from the Milan criteria (Milan guidelines) to the University of California, San Francisco (UCSF) criteria (University of California, San Francisco, USA) which reduced the restrictions on liver transplantation. For example, for a single liver cancer tumor, the size changed from <5 cm to <6.5 cm; in the case of multiple liver cancer tumors, the number and size of tumors was adjusted from more than 3 and <3 cm to <4.5 cm with the total tumor diameter less than 8 cm. The change in liver transplant standards and guidelines made liver transplantation available to more liver cancer patients, but it may also have had the effect of causing transplantation to occur in sicker patients, who were at increased risk of recurrence of hepatocellular carcinoma and death.

In summary, this study was the first to examine surgeon cumulative service volume in liver transplantation at a population level and to describe its association with 1-year post-transplant survival as a representation of the surgical learning curve. The results demonstrated that 1-year survival after liver transplantation tended to increase with higher surgeon service volume (relative volume). When surgeons had accumulated approximately 41–60 liver transplant cases, patient 1-year survival reached approximately 85% and became more stable.

It should be emphasized that these findings demonstrate an association rather than a causal relationship, and the observed service volume range should not be interpreted as a definitive threshold or prescriptive standard. Given the retrospective design and the use of an administrative database, the results should be viewed as descriptive and hypothesis-generating.

Accordingly, the clinical relevance of this study lies in providing population-level evidence that may complement existing clinical risk assessments and facilitate further discussion regarding the role of surgeon experience in liver transplantation outcomes. Before any policy-level recommendations or structural modifications to transplant programs are considered, further studies incorporating comprehensive clinical registries, center-level adjustment, and advanced statistical modeling are warranted.

## 5. Limitation

This study has several limitations. First, the Charlson Comorbidity Index (CCI) was used to represent baseline health status; however, it may not fully reflect the acute physiological severity of liver transplant candidates. Second, the database covered the period from 2005–2013 and may not entirely represent current transplantation practices, although the volume–outcome relationship is likely to remain valid over time. Third, information on graft source (living or deceased donor) was unavailable, but both types are reimbursed under the same procedural code and follow similar management in Taiwan’s National Health Insurance system, minimizing potential bias. Finally, several clinical and graft-related factors—including MELD score, portal hypertension, hepatocellular carcinoma, organ function, and graft-to-recipient ratio—were not recorded, and inter-institutional heterogeneity could not be fully adjusted. These limitations should be considered when interpreting the association between surgeon service volume and patient survival.

## Figures and Tables

**Table 1:** Distribution of Characteristics of the Study Subjects.

Variable	N	%
Total	3233	100
1-year survival after surgery		
Dead	459	14.20
Alive	2774	85.80
Surgeon’s relative service volume (cases)		
Low (≤31)	811	25.09
Medium (32–306)	1611	49.82
High (≥307)	811	25.09
Surgeon’s cumulative service volume (cases)		
≤20	619	19.15
21–40	320	9.90
41–60	249	7.70
61–80	193	5.97
81–100	123	3.80
101–120	124	3.84
121–140	119	3.68
141–160	118	3.65
161–180	125	3.87
181–200	105	3.25
201–230	104	3.22
231–260	88	2.72
261–300	117	3.62
301–400	297	9.19
401–500	210	6.50
≥501	322	9.96
Patient sex		
Male	2299	71.11
Female	934	28.89
Patient age (years)		
<20	250	7.73
20–34	132	4.08
35–44	360	11.14
55–64	1181	36.53
≥65	185	5.72
Monthly salary (USD)		
<564	601	18.59
564–744	1151	35.60
745–940	298	9.22
941–1186	324	10.02
1187–1496	488	15.09
≥1497	371	11.48
Charlson Comorbidity Index		
0	701	21.68
1–2	1391	43.03
≥3	1141	35.29
Urbanization of residence area		
Level 1	824	25.49
Level 2	1020	31.55
Level 3	568	17.57
Level 4	498	15.40
Level 5	65	2.01
Level 6	118	3.65
Level 7	140	4.33
Whether the surgeon changed the practice hospital		
Yes	8	0.25
No	3225	99.75
Hospital accreditation level		
Medical center	3139	97.09
Regional hospital	94	2.91
Hospital ownership		
Public hospital	1027	31.77
Non-public hospital	2206	68.23
Year of transplantation		
2005	177	5.47
2006	210	6.50
2007	254	7.86
2008	325	10.05
2009	347	10.73
2010	429	13.27
2011	503	15.56
2012	512	15.84
2013	476	14.72
Year after surgery	Alive (N)	Survival rate (%)
First year	2774	85.80
Second year	2660	82.25
Third year	2595	80.27
Fourth year	2555	79.03
Fifth year	2527	78.16

USD: United States dollar.

**Table 2:** Bivariate analysis of factors associated with 1-year survival status of liver transplant patients.

Variable	Survival, N	Survival (%)	*p*-Value
Total	2774	85.80	
Surgeon’s relative service volume (cases)			<0.001
Low (≤31)	579	71.39	
Medium (32–306)	1422	88.27	
High (≥307)	773	95.31	
Surgeon’s cumulative service volume (cases)			<0.001
≤20	439	70.92	
21–40	241	75.31	
41–60	217	87.15	
61–80	164	84.97	
81–100	105	85.37	
101–120	113	91.13	
121–140	107	89.92	
141–160	103	87.29	
161–180	117	93.60	
181–200	101	96.19	
201–230	92	88.46	
231–260	76	86.36	
261–300	110	94.02	
301–400	276	92.93	
401–500	198	94.29	
≥501	315	97.83	
Patient sex			0.823
Male	1974	85.86	
Female	800	85.65	
Patient age			0.350
<20	216	86.40	
20–34	114	86.36	
35–44	315	87.50	
45–54	980	87.11	
55–64	995	84.25	
≥65	154	83.24	
Monthly salary (USD)			0.148
<564	499	83.03	
564–744	983	85.40	
745–940	255	85.57	
941–1186	283	87.35	
1187–1496	430	88.11	
≥1497	324	87.33	
Charlson Comorbidity Index			0.001
0	621	88.59	
1–2	1208	86.84	
≥3	945	82.82	
Urbanization of residence area			0.522
Level 1	697	84.59	
Level 2	888	87.06	
Level 3	493	86.80	
Level 4	426	85.54	
Level 5	53	81.54	
Level 6	101	85.59	
Level 7	116	82.86	
Whether the surgeon changed the practice hospital			0.053
Yes	5	62.50	
No	2769	85.86	
Hospital accreditation level			0.044
Medical center	2700	86.01	
Regional hospital	74	78.72	
Hospital ownership			0.026
Public hospital	862	83.93	
Non-public hospital	1912	86.67	
Year of transplantation			<0.001
2005	144	81.36	
2006	163	77.62	
2007	202	79.53	
2008	277	85.23	
2009	305	87.90	
2010	372	86.71	
2011	434	86.28	
2012	447	87.30	
2013	430	90.34	

USD: United States dollars.

**Table 3:** Effects and associated risk factors of different surgeon service volumes on mortality of patients 1 year after liver transplantation.

Variable	Adjusted Model 1				Adjusted Model 2			
	HR	95%C.I.		*p*-Value	HR	95%C.I.		*p*-Value
Surgeon’s relative service volume (cases)								
Low (≤31)	1.00							
Medium (32–306)	0.35	0.28	0.43	<0.001				
High (≥307)	0.13	0.09	0.19	<0.001				
Surgeon’s cumulative service volume (cases)								
≤20					1.00			
21–40					0.83	0.63	1.09	0.179
41–60					0.40	0.27	0.59	<0.001
61–80					0.46	0.31	0.69	<0.001
81–100					0.46	0.28	0.75	0.002
101–120					0.25	0.14	0.47	<0.001
121–140					0.28	0.15	0.51	<0.001
141–160					0.37	0.21	0.63	<0.001
161–180					0.18	0.09	0.38	<0.001
181–200					0.12	0.04	0.31	<0.001
201–230					0.34	0.19	0.62	<0.001
231–260					0.36	0.20	0.65	0.001
261–300					0.14	0.07	0.31	<0.001
301–400					0.19	0.12	0.30	<0.001
401–500					0.16	0.09	0.31	<0.001
≥501					0.06	0.03	0.12	<0.001
Patient sex								
Male	1.00				1.00			
Female	0.95	0.77	1.17	0.639	0.98	0.79	1.21	0.848
Patient age								
<20	1.00				1.00			
20–34	0.88	0.49	1.59	0.680	0.83	0.46	1.49	0.526
35–44	0.81	0.50	1.32	0.399	0.75	0.46	1.21	0.239
45–54	0.90	0.59	1.37	0.609	0.84	0.55	1.29	0.427
55–64	1.12	0.74	1.70	0.593	1.05	0.69	1.59	0.835
≥65	1.23	0.72	2.09	0.451	1.13	0.66	1.93	0.653
Monthly salary (USD)								
<564	1.00				1.00			
564–744	0.89	0.70	1.15	0.385	0.92	0.71	1.18	0.505
745–940	0.84	0.59	1.21	0.349	0.85	0.59	1.23	0.391
941–1186	0.77	0.53	1.11	0.160	0.77	0.53	1.11	0.165
1187–1496	0.77	0.55	1.07	0.121	0.80	0.57	1.11	0.183
≥1497	0.82	0.58	1.16	0.264	0.87	0.61	1.23	0.420
Charlson Comorbidity Index								
0	1.00				1.00			
1–2	1.21	0.90	1.62	0.204	1.21	0.90	1.63	0.201
≥3	1.52	1.13	2.05	0.006	1.54	1.14	2.08	0.005
Urbanization of residence area								
Level 1	1.00				1.00			
Level 2	0.92	0.72	1.18	0.506	0.91	0.71	1.17	0.456
Level 3	0.87	0.65	1.16	0.337	0.87	0.65	1.16	0.336
Level 4	0.94	0.70	1.27	0.703	0.94	0.69	1.26	0.657
Level 5	1.27	0.70	2.33	0.431	1.28	0.70	2.34	0.427
Level 6	0.73	0.44	1.24	0.244	0.76	0.45	1.28	0.303
Level 7	1.21	0.77	1.89	0.402	1.16	0.74	1.82	0.510
Whether the surgeon changed the practice hospital								
Yes	1.00				1.00			
No	0.75	0.23	2.41	0.627	0.77	0.24	2.50	0.668
Hospital accreditation level								
Medical center	1.00				1.00			
Regional hospital	0.64	0.40	1.03	0.064	0.62	0.39	1.00	0.051
Hospital ownership								
Public hospital	1.00				1.00			
Non-public hospital	1.09	0.88	1.34	0.443	1.18	0.95	1.46	0.133
Year of transplantation								
2005	1.00				1.00			
2006	1.16	0.74	1.82	0.516	1.12	0.71	1.76	0.637
2007	1.20	0.78	1.87	0.410	1.24	0.80	1.94	0.343
2008	0.89	0.57	1.40	0.617	0.90	0.57	1.41	0.637
2009	0.77	0.48	1.21	0.257	0.73	0.46	1.16	0.183
2010	0.97	0.62	1.50	0.876	0.97	0.62	1.51	0.885
2011	0.98	0.64	1.50	0.938	1.03	0.67	1.58	0.897
2012	1.11	0.72	1.71	0.637	1.07	0.68	1.66	0.781
2013	0.88	0.56	1.40	0.592	0.89	0.56	1.43	0.635

Event = death; USD: United States dollars.

## Data Availability

The data used in this study are available from the National Health Insurance Research Database and the Cause of Death Database, Taiwan. Access to these databases is restricted and requires approval from the Ministry of Health and Welfare, Taiwan.

## Abbreviations

The following abbreviations are used in this manuscript:

CCI	Charlson Comorbidity Index
MELD	Model for End-Stage Liver Disease
